# Differential psychosocial consequences between male and female workers who have suffered an amputation in an occupational accident

**DOI:** 10.23938/ASSN.1090

**Published:** 2024-11-14

**Authors:** Rubén Nevado, Alfonso Arteaga, Javier Fernández-Montalvo

**Affiliations:** 1 Public University of Navarre Department of Health Sciences Pamplona Spain; 2 Navarre Institute of Health Research (IdisNa) Pamplona Spain

**Keywords:** Amputation, Occupational Accident, Psychosocial Consequences, Gender, Assessment, Amputación, Accidente Laboral, Consecuencias Psicosociales, Género, Evaluación

## Abstract

**Background:**

The main goals of this study are to determine the sociodemographic, occupational and psychosocial characteristics and type of amputation of people affected by occupational amputations in Navarre, Spain, and to analyse the differing characteristics based on sex.

**Methods:**

People affected by occupational amputations in Navarra between January 2000 and December 2019 were identified by the Public and Work Health Institute of Navarre. Sociodemographic, amputation, psychopathological (Symptom Checklist, SCL-90-R; Severity Posttraumatic Stress Disorder Scale-Revised, EGS-R), maladjustment (Maladjustment Scale), pain (Numeric Pain Rating Scale) and suicide (Columbia Scale Screening for Suicidal Ideation, C-SSRS) characteristics were assessed.

**Results:**

Of the 557 workers identified, 80 were included in the study. The results showed the presence of relevant psychosocial repercussions with a moderately high level of psychopathological symptoms, a Post-Traumatic Stress Disorder (PTSD) pre-valence rate of 10%, re-experimentation as the most relevant symptom, and labour and leisure as the most affected areas. Sex differences were found in PTSD re-experimentation symptoms (higher in women) and leisure maladjustment (higher in men). No sex differences were found in the remaining variables studied.

**Conclusions:**

This study demonstrates the relevance of psychosocial consequences in workers with amputations. Research on this topic is necessary due to the scarcity of studies conducted to date.

## RESUMEN

**Fundamento:**

Los principales objetivos de este estudio son determinar las características sociodemográficas, tipo de amputación, ocupacionales y psicosociales de las personas afectadas por amputaciones ocupacionales en Navarra, España, y analizar las diferentes características en función del sexo.

**Método:**

El Instituto de Salud Pública y Laboral de Navarra identificó a los trabajadores que habían sufrido una amputación en un accidente laboral en Navarra entre enero de 2000 y diciembre de 2019. Se evaluaron las características sociodemográficas, de la amputación, psicopatológicas (Listado de Síntomas, SCL-90-R; Escala de Gravedad de Síntomas Revisada del Trastorno de Estrés Postraumático, EGS-R), inadaptación (Escala de Inadaptación), dolor (Escala Numérica de Valoración del Dolor) y suicidio (Escala Columbia para Evaluar el Riesgo de Suicidio, C-SSRS).

**Resultados:**

De los 557 trabajadores identificados, 80 fueron incluidos en el estudio. Los resultados mostraron la presencia de repercusiones psicosociales relevantes con un nivel moderadamente alto de síntomas psicopatológicos, una tasa de prevalencia de TEPT del 10%, la reexperimentación como síntoma más relevante y el trabajo y el ocio como áreas más afectadas. Se encontraron diferencias por sexo en los síntomas de reexperimentación del TEPT (mayores en mujeres) y en la inadaptación del ocio (mayores en hombres). No se encontraron diferencias por sexo en el resto de variables estudiadas.

**Conclusiones:**

Este estudio demuestra la relevancia de las consecuencias psicosociales en trabajadores con amputaciones. Es necesaria más investigación sobre este tema dado el escaso número de estudios desarrollados hasta la fecha.

## INTRODUCTION

Occupational accidents are one of the main causes of disability in workers^1^ and, more specifically, of amputations^2^. Traumatic origin is the second leading cause of amputation in developed countries, after amputations due to disease, and it is the main cause in nondeveloped countries^3^. In a study by McDonald et al.^4^, 57.7 million people in the world had undergone some type of limb amputation due to traumatic causes. In Spain, 0.18% of the population has suffered an amputation^5^.

People with traumatic work-related amputations are a special group within people who have undergone an amputation, as they present suddenly^6^. This type of amputation causes significant changes in different areas of the affected person’s life^7^, mainly in the psychological domain^8^. These changes are more relevant during the first two years following the amputation^9^.

Most of the studies about psychological consequences of traumatic amputations are focused on depression and anxiety^10^, symptoms of posttraumatic stress disorder^11^, body image problems^12^, pathological grief^13^ and phantom limb pain^14^. However, there is a significant absence of studies focused on sudden amputations in the workplace. The scarce studies^1^^,^^15^^-^^17^ do not focus on psycholo-gical consequences. Only the study of Cheung et al.^2^ has identified posttraumatic stress disorder (PTSD) and depression in people who have suffered amputations in an occupational accident. Therefore, the specific psychological consequences for this type of workers are unknown.

With regard to anxiety and depression, people with amputations present high levels of symptoms during the first years after amputation, but these decrease in the long term^9^. Some studies show relevant prevalence rates of anxiety in this population: 29.9% in a study by Atherton and Robertson^1^^,^^8^ and 34% in the study of Desmond and MacLachlan^19^, both above the 12.6% found in the general population^20^. However, in other studies, no differences from the general population’s anxiety levels have been found^21^^,^^22^. Regarding depressive symptoms, a great variability in prevalence rates has been found, ranging from 15% to 80.7%^23^^,^^24^. Therefore, the results found are heterogeneous and prevent us from reaching solid conclusions.

Some attempts to assess the presence of posttraumatic symptoms in people with amputations have been conducted, most of them focused on war veterans, with PTSD. ranging from 24.6% to 36%^25^^,^^26^.

Despite the few studies conducted about suicidal behaviour in persons who have suffered an amputation, the rates found are significant: the prevalence of suicidal ideation ranges from 29.2% to 36%^27^^,^^28^, and suicide attempts range from 12% to 27.5%^27^^,^^29^.

People who have suffered an amputation usually present with phantom limb pain. This phenomen affects between 50% and 85% of people who suffer amputation^14^.

Beyond the psychological consequences mentioned, some studies have focused on the repercussions of amputations on the quality of life and general mental health. Jiménez et al.^30^ found that 53.5% of people with amputations had a poor quality of life and 82.1% experienced mental health problems. However, only 28.6% had attended psychological therapy.

From another perspective, several studies show that a good adaptation to daily life is one of the main variables related to a good quality of life in people who have had amputations^31^. These studies have been conducted in the field of partner relationships^32^, social support^33^^,^^34^ and leisure^12^^,^^35^.

In any case, the scarcity of studies on the psychological consequences of occupational amputations and the lack of homogeneity of the data found show the need to continue developing studies on this subject. Few studies have been carried out with specific samples of workers who have suffered an amputation at work. Additionally, the studies do not differentiate between different types of amputations, level of severity, or whether the amputation is due to a work-related accident or another type of traumatic amputation.

On the other hand, the gender perspective has hardly been considered. The study of Ali and Haider^36^ showed that men presented more signs of maladjustment than women. However, there are no further studies focusing on the differences between men and women. Probably, the jobs where the most amputations occur have traditionally been associated with men. Future studies should adopt a gender perspective in the analysis of the consequences of suffering a work amputation.

Therefore, the main goals of this study are, first, to determine the sociodemographic, occupational and psychosocial characteristics and the type of amputation of people affected by occupational amputations in Navarre, Spain. Second, to analyse the differential characteristics from a gender perspective. The main hypotheses are that people with amputations will show relevant psychosocial consequences (more psychopathological symptoms, higher rates of posttraumatic symptoms, and worse adjustment to daily life) and that differences between men and women will be found (men will show more signs of maladjustment than women; and women will present more psychosocial symptoms than men).

## MATERIALS AND METHODS

### Design

This is a descriptive, ex post facto, retrospective study carried out in Navarre, Spain, with workers who suffered an amputation in an occupational accident between January 2000 and December 2019.

### Participants

The target population was identified by the Public and Work Health Institute of Navarre (Instituto de Salud Publica y Laboral de Navarra-ISPLN). The inclusion criteria were as follows: a) having suffered an amputation in an occupational accident in Navarre; b) being sufficiently proficient in the Spanish language to complete the assessment tools; and c) signing the informed consent to participate in the study.

### Instruments

The self-reported *Symptom Checklist* (SCL-90-R)^37^^)^ assesses psychopathological symptoms. It is composed of 90 items, which are answered on a five-point Likert scale from 0 (*nothing*) to 4 (*extremely*). This tool aims to reflect the symptoms of psycholo-gical distress. It consists of nine primary symptom dimensions (somatization, obsession-compulsion, interpersonal sensitivity, depression, anxiety, hostility, phobic anxiety, paranoid ideation, and psychoticism), and three global indices of severity: the Global Severity Index (GSI), which reflects overall symptom severity, the Positive Symptom Distress Index (PSDI), which indicates symptom intensity, and the Positive Symptom Total (PST), which includes the number of items answered with a score different from 0. The internal consistency ranges from .070 to 0.90. In this study, the percentiles of each dimension were considered.

The *Severity Posttraumatic Stress Disorder Scale - Revised* (EGS-R)^38^ is a Spanish semistructured interview that evaluates the presence of PTSD and the severity of symptoms. It is based on the diagnostic criteria of the DSM-5 and is composed of two parts: a) exposure to a traumatic event, and b) severity and frequency of PTSD symptoms. It consists of 21 items scored from 0 (*nothing/never*) to 3 (*extremely/5 or more times a week*) on a four-point Likert scale. In addition, six items valorise the interference of trauma in different areas of daily life. The cut-off point of the total scale is 20. Moreover, the scale contains four subscales: re-experimentation (cut-off point = 3), behavioural/cognitive avoidance (cut-off point = 3), cognitive alterations and negative mood (cut-off point = 5) and increased activation and psychophysiological reactivity (cut-off point = 5). The internal consistency is 0.91.

The *Maladjustment Scale*^39^ reflects the extent to which each patient’s situation affects various areas of everyday life: work or school, social life, leisure, partner relationships, family life, and global situations. This instrument includes six items ranging from 0 (*nothing*) to 5 (*a lot*) on a Likert scale. The total scale range is 0-30. The cut-off point revealing a significant maladjustment is two points for each area and 12 points for the full scale. The internal consistency is 0.94.

The *Numeric Pain Rating Scale*^40^ is the tool most commonly used to capture the patient’s level of pain. It is an 11-point scale that assesses patients’ perception of pain. The scale is anchored from 0 (*no pain*) to 10 (*worst imaginable pain*). Patients rate their level of pain in the last week.

The *Columbia Scale Screening for Suicidal Ideation* (C-SSRS)^41^ is a semistructured interview that reflects the severity and intensity of ideation, suicidal attempts and lethality. This scale was recently validated with a Spanish sample^42^. The current study used the screening version that assesses five types of ideation of increasing gravity with an ordinal scale of five points from 1 (*wish to die*) to 5 (*suicidal ideation with specific plan and intention*) and one additional question regarding suicidal behaviour.

### Procedure

The Ethics Committee of Universidad Pública de Navarra and ISPLN approved the protocol for this study according to ethical guidelines and Spanish data protection laws.

ISPLN sent a letter to all workers who had suffered an amputation in an occupational accident during the duration of the study. In this letter, they were informed of the goals of the study and were requested to participate voluntarily. Subsequently, they were contacted by telephone to confirm or reject their participation. Informed consent was obtained from all individual participants included in the study.

All patients who participated were interviewed by the same clinical psychologist, who was specialized in the evaluation and treatment of the psychological consequences of amputation. The assessment of the sample was carried out in a two-hour session in which sociodemographic data, accident/amputation variables and psychopathological symptoms were evaluated.

### Data Analysis

Descriptive analyses were conducted for all variables (frequencies or mean with standard deviation (SD). In the bivariate analyses, comparisons between groups were performed using χ^2^ or Student’s t test for independent samples, depending on the nature of the variables analysed. Effect sizes (Cohen’s d or Phi coefficient) were provided for all of the analyses. A difference of p <0.05 was considered significant. All statistical analyses were performed using SPSS (vs. 27.0) software.

## RESULTS

**Figure 1:**
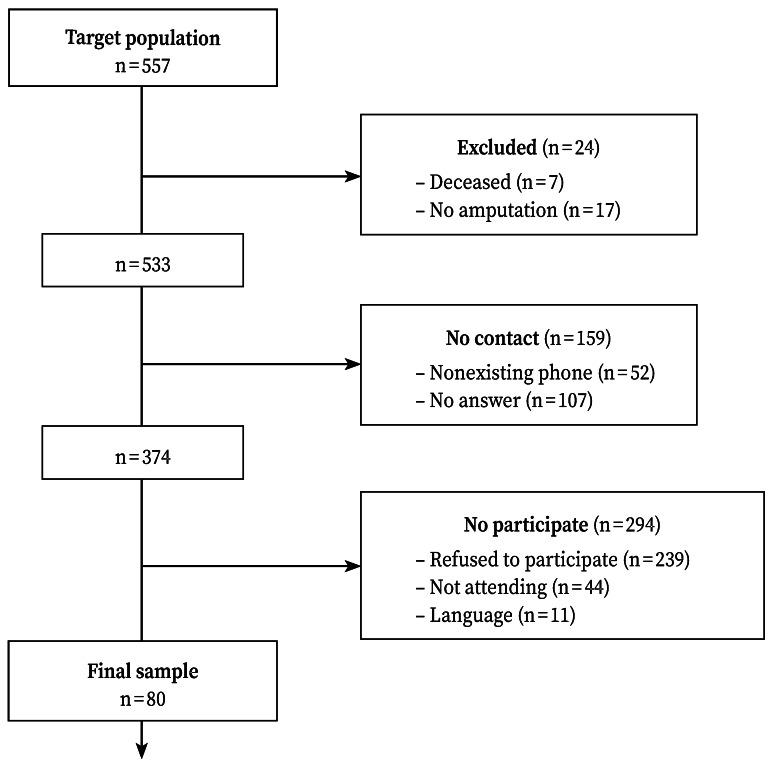
Flow chart.

The Public and Work Health Institute of Navarre (Instituto de Salud Publica y Laboral de Navarra-ISPLN) identified the 557 workers who had undergone an amputation due to an occupational accident in Navarre, Spain, between 2000 and 2019. The reasons for which 477 persons were not included in the study are shown in the flow chart (Figure 1). Specifically, 7 had died, 17 did not actually suffer an amputation, 159 could not be contacted, 283 refused to participate or did not attend the assessment session, and 11 did not speak Spanish.

According to these criteria, 80 subjects were included in the study; of these, 83.8% were men (*n* = 67), and 16.2% were women (*n* = 13).

### Sociodemographic characteristics

Regarding sociodemographic variables, the mean age of the participants was 48.8 years. Most of the sample were Spanish men who were married, had secondary degrees and were employed. The only variable with statistically significant differences between men and women was educational level. Most women had primary degrees, while most men had secondary degrees. No differences were observed in the rest of the variables (Table 1).

**Table 1:** Sociodemographic characteristics, global and compared by sex

	Total n (%)	Men n (%)	Women n (%)	p (χ^2^)	Effect size (Phi)
Age*	46.8 (9.9)	47.5 (9.7)	43.7 (11.2)	0.218	0.374
Nationality				0.121	0.173
Spanish	67.0 (83.8)	58.0 (86.8)	9.0 (69.2)		
Foreign	13.0 (16.2)	9.0 (13.4)	4.0 (30.8)		
Marital status				0.703	0.133
Married	57.0 (71.3)	49.0 (73.1)	8.0 (61.5)		
Single	11.0 (13.8)	8.0 (11.9)	3.0 (23.1)		
Separated/Divorced	11.0 (13.8)	9.0 (13.4)	2.0 (15.4)		
Widower	1.0 (1.3)	1.0 (1.5)	0.0 (0)		
Educational level				0.035	0.329
No studies	2.0 (2.5)	2.0 (3)	0.0 (0)		
Primary	21.0 (26.3)	14.0 (20.9)	7.0 (53.8)		
Secondary	55.0 (68.8)	50.0 (74.6)	5.0 (38.5)		
University	2.0 (2.5)	1.0 (1.5)	1.0 (7.7)		
Employment situation				0.488	0.077
Employed	69.0 (86.3)	57.0 (85.1)	12.0 (92.3)		
Unemployed	11.0 (13.8)	10.0 (14.9)	1.0 (7.7)		

*: Mean (standard deviation), compared by Student-t test and effect size was calculated according to Cohen’s d.

### Accident and amputation characteristics

The mean time elapsed since the accident that caused the amputation was 8.3 years. In most cases, the amputation was caused by a machine and affected the upper half and the left side of the body. The main segment amputated was a finger. No significant differences in variables related to amputation were found between men and women (Table 2).

**Table 2:** Comparison of variables related to the accident and amputation between men and women

	Total n (%)	Men n (%)	Women n (%)	p (χ^2^)	Effect size (Phi)
Time elapsed (years)*	8.3 (5.2)	8.5 (5.3)	7.4 (4.5)	0.491	0.211
Aetiology					
Machine	62.0 (77.5)	53.0 (79.1)	9.0 (69.2)	0.390	0.153
Material handling	16.0 (20)	13.0 (19.4)	3.0 (23.1)		
*In itinere*	2.0 (2.5)	1.0 (1.5)	1.0 (7.7)		
Level					
Upper half	76.0 (95)	63.0 (94)	13.0 (100)	0.366	0.101
Lower half	4.0 (5)	4.0 (6)	0.0 (0)		
Laterality					
Left	44.0 (55)	36.0 (53.7)	8.0 (61.5)	0.813	0.072
Right	35.0 (43.8)	30.0 (44.8)	5.0 (38.5)		
Both side	1.0 (1.2)	1.0 (1.5)	0.0 (0)		
Body segment					
Eye	2.0 (2.5)	2.0 (3)	0.0 (0)	0.692	0.195
Arm	1.0 (1.2)	1.0 (1.5)	0.0 (0)		
Hand	2.0 (2.5)	1.0 (1.5)	1.0 (7.7)		
Finger	71.0 (88.8)	59.0 (88.1)	12.0 (92.3)		
Leg	2.0 (2.5)	2.0 (3)	0.0 (0)		
Toe	2.0 (2.5)	2.0 (3)	0.0 (0)		

*: Mean (standard deviation), compared by Student-t test and effect size was calculated according to Cohen’s d.

### Psychopathological and maladjustment consequences

The results of the SCL-90-R showed a moderately high level of psychopathological symptoms, without significant differences between men and women (Table 3).

Posttraumatic stress disorder was present in 10% of the sample, without gender differences. The most PTSD relevant symptom was re-experimentation, with more women affected than men. No significant differences in the remaining PTSD symptoms were found.

**Table 3:** Psychopathological symptoms, posttraumatic stress disorder and maladjustment, global and compared by sex

Score - Participants above cut-off point	Total	Men	Women	p	Effect size
				t-test	Cohen’s d
				χ^2^	Phi
*Psychopathological symptoms (SCL-90-R)*					
Somatization					
Mean (SD)	72.3 (24.7)	73.1 (26.1)	68.6 (15.9)	0.557	0.176
n (%)	46.0 (57,5)	41.0 (61.2)	5.0 (38.5)	0.129	0.170
Obsession-compulsion					
Mean (SD)	59.5 (32.4)	56.7 (34.5)	74.3 (22)	0.072	0.544
n (%)	37.0 (46,3)	29.0 (43.3)	8.0 (61.5)	0.227	0.135
Interpersonal sensitivity					
Mean (SD)	60.0 (32.2)	58.1 (33)	69.5 (27)	0.243	0.355
n (%)	35.0 (43,8)	28.0 (41.8)	7.0 (53.8)	0.423	0.090
Depression					
Mean (SD)	52.5 (31.3)	50.8 (31.3)	61.5 (30.7)	0.257	0.345
n (%)	25.0 (31,3)	18.0 (26.9)	7.0 (53.8)	0.055	0.215
Anxiety					
Mean (SD)	53.4 (32.3)	51.7 (33.2)	62.2 (26.4)	0.289	0.323
n (%)	33.0 (41,3)	27.0 (40.3)	6.0 (46.2)	0.695	0.044
Hostility					
Mean (SD)	50.9 (31.6)	50.7 (31.8)	52.2 (31.8)	0.881	0.045
n (%)	23.0 (28,8)	18.0 (26.9)	5.0 (38.5)	0.398	0.095
Phobic anxiety					
Mean (SD)	38.3 (37.9)	35.9 (37.6)	50.6 (38.4)	0.203	0.387
n (%)	20.0 (25)	14.0 (20.9)	6.0 (46.2)	0.054	0.215
Paranoid ideation					
Mean (SD)	57.5 (32.1)	55.9 (32)	66.0 (32.4)	0.301	0.315
n (%)	31.0 (38,8)	23.0 (34.3)	8.0 (61.5)	0.065	0.206
Psychoticism					
Mean (SD)	51.1 (33.7)	48.0 (34.8)	67.2 (22.2)	0.059	0.571
n (%)	27.0 (33,8)	22.0 (32.8)	5.0 (38.5)	0.695	0.044
GSI					
Mean (SD)	62.5 (30.1)	60.8 (31.1)	70.9 (23.3)	0.275	0.332
n (%)	37.0 (46,3)	29.0 (43.3)	8.0 (61.5)	0.227	0.135
PST					
Mean (SD)	64.9 (29.9)	62.9 (31)	75.2 (21.6)	0.175	0.412
n (%)	41.0 (51,3)	33.0 (49.3)	8.0 (61.5)	0.417	0.091
PSDI					
Mean (SD)	50.3 (27.8)	50.2 (28.5)	51.0 (24.9)	0.922	0.030
n (%)	18.0 (22,5)	16.0 (23.9)	2.0 (15.4)	0.502	0.075
*Posttraumatic stress disorder (EGS-R)*					
Re-experimentation					
Mean (SD)	2.2 (2.9)	2.1 (3.1)	2.7 (1.8)	0.517	0.198
n (%)	24.0 (30)	17.0 (25.4)	7.0 (53.8)	0.040	0.229
Behavioural/cognitive avoidance					
Mean (SD)	1.0 (1.6)	1.1 (1.7)	0.9 (1.1)	0.671	0.129
n (%)	13.0 (16.3)	12.0 (17.9)	1.0 (7.7)	0.361	0.102
Cognitive alterations and negative mood					
Mean (SD)	1.8 (2.9)	1.8 (3)	1.7 (2.3)	0.924	0.029
n (%)	10.0 (12.5)	8.0 (11.9)	2.0 (15.4)	0.731	0.038
Increased activation and psychophysiological reactivity					
Mean (SD)	2.4 (2.8)	2.4 (3)	2.7 (2)	0.700	0.117
n (%)	15.0 (18.8)	12.0 (17.9)			0.049
Total score PSTD					
Mean (SD)	7.4 (8.9)	7.3 (9.5)	7.9 (5.2)	0.823	0.068
n (%)	8.0 (10)	8.0 (11.9)	0.0 (0)	0.189	0.147
*Maladjustment*					
Labour					
Mean (SD)	1.7 (1.6)	1.8 (1.6)	1.6 (1.7)	0.784	0.083
n (%)	39.0 (48.8)	32.0 (47.8)	7.0 (53.8)	0.688	0.045
Social					
Mean (SD)	0.7 (1.2)	0.8 (1.2)	0.4 (0.8)	0.288	0.323
n (%)	16.0 (20)	15.0 (22.4)	1.0 (7.7)	0.225	0.136
Leisure					
Mean (SD)	1.4 (1.6)	1.5 (1.6)	0.5 (1)	0.021	0.629
n (%)	34.0 (42.5)	32 (47.8)	2.0 (15.4)	0.031	0.242
Partner					
Mean (SD)	0.5 (1.2)	0.6 (1.2)	0.2 (0.6)	0.320	0.303
n (%)	13.0 (16.3)	12 (17.9)	1.0 (7.7)	0.361	0.102
Family					
Mean (SD)	0.5 (1.1)	0.5 (1.1)	0.5 (1)	0.924	0.029
n (%)	11.0 (13.8)	9 (13.4)	2.0 (15.4)	0.852	0.021
General					
Mean (SD)	1.4 (1.4)	1.4 (1.4)	1.2 (1.3)	0.573	0.172
n (%)	32.0 (40)	26 (38.8)	6.0 (46.2)	0.621	0.055
Total score maladjustment					
Mean (SD)	6.2 (6.4)	6.5 (6.8)	4.3 (4)	0.262	0.341
n (%)	10.0 (12.5)	11 (16.4)	0.0 (0)	0.116	0.176

SD: standard deviation; SCL-90-R: Self-reported Symptom Checklist (percentiles); EGS-R: Severity Posttraumatic Stress Disorder Scale - Revised.

With regard to maladjustment, the most affected areas were labour and leisure. Significant gender differences were found in leisure, with more men than women being affected.

### Pain, phantom member, suicide ideation and treatments

Almost half of the sample presented with severe (18.8%) or moderate (30%) intensity of pain (Table 4). Moreover, phantom limb syndrome affected 66.7% of participants. No significant gender differences for either variable were found.

Regarding suicide ideation, 2.5% of the sample had thought of killing themselves, but no suicide attempts were observed. Immediately after the accident, most of the participants (98.8%) received pharmacological treatment, and 17.5% received psychological treatment. No significant differences between groups were observed.

**Table 4:** Pain, suicide and treatments, global and compared by sex

	Total n (%)	Men n (%)	Women n (%)	p (χ^2^)	Effect size (Phi)
Pain intensity*	3.8 (2.9)	3.6 (2.8)	5.1 (3.3)	0.083	0.525
Pain scale^a^				0.527	0.167
without pain (0)	15.0 (18.8)	14.0 (20.9)	1.0 (7.7)		
soft (1-3)	26.0 (32.5)	22.0 (32.8)	4.0 (30.8)		
moderate (4-6)	24.0 (30)	20.0 (29.9)	4.0 (30.8)		
severe (7-10)	15.0 (18.8)	11.0 (16.4)	4.0 (30.8)		
Phantom member^b^	52.0 (66.7)	42.0 (64.6)	10.0 (76.9)	0.738	0.390
Suicide ideation^c^	2.0 (2.5)	1.0 (1.5)	1.0 (7.7)	0.190	0.146
Pharmacological treatment					
after the accident	79.0 (98.8)	66.0 (98.5)	13.0 (100)	0.658	0.050
at the time of the study	27.0 (33.8)	20.0 (29.9)	7.0 (53.8)	0.094	0.187
Psychological treatment					
after the accident	14.0 (17.5)	13.0 (19.4)	1.0 (7.7)	0.309	0.114
at the time of the study	4.0 (5)	3.0 (4.5)	1.0 (7.7)	0.626	0.054

*: Mean (standard deviation), compared by Student-t test and effect size calculated according to Cohen’s d; a: Numeric Pain Rating Scale; b: two persons were excluded because of presenting eye amputation; c: Columbia Scale Screening for Suicidal Ideation (C-SSRS).

## DISCUSSION

In this study, a specific sample of workers who had suffered an amputation derived from an occupational accident was studied. The results support the presence of relevant psychosocial repercussions in workers with amputations. Moreover, a specific contribution of this study is that this is the first study related to amputations that includes the comparison between men and women in the analyses of the results.

The participants showed a moderately high level of psychopathological symptoms, a PTSD prevalence rate of 10%, with re-experimentation as the most relevant symptom and with labour and leisure as the most affected areas. Therefore, the first hypothesis of the study is met.

Most of the amputations in this study affected the upper half and the left side of the body, with the finger being the main body part amputated. These results are slightly different from previous studies carried out with traumatic amputations that were not necessarily derived from work accidents. In a study by McDonald et al.^4^, for example, 42.8% of the amputations studied affected a lower limb, and 38.7% affected an upper limb. In contrast, in our study, 95% of amputations affected the upper half of the body. This higher rate of upper limbs affected is probably related to the occupational aetiology of the accident, as most labour activities require the use of hands and arms. When men and women were compared, no gender differences were found in the nature of the amputations. However, no previous studies comparing these gender-related results have been found.

Regarding psychopathological symptoms, clinical anxiety affected 41.3% of the workers with amputations. This rate is higher than those found in previous studies with amputations not necessarily derived from work accidents^18^^,^^19^. The rate of clinically relevant symptoms of depression found in our study (31.3%) is in the same range as those of previous studies^23^^,^^24^. Regardless, beyond the results for anxiety and depression, the main clinically relevant psychopathological symptom found in this study was somatization, which affected 57.5% of workers with amputations. Although there are no previous studies with which to compare this result of somatization, the presence of physical symptoms in persons who have suffered a traumatic amputation is common. Further studies are needed to differentiate physical symptoms directly related to amputation from specific somatization symptoms, as items of the SCL-90-R may conflate psychopathological symptoms with the usual physical symptoms of amputations. Regarding the gender perspective, contrary to what was hypothesized, no differences between men and women in psychopathological symptoms were found.

On the other hand, the rate of PTSD in this study was 10%. Although there are no comparative studies with similar samples, this rate is clearly lower than those found in other studies with war veterans who have undergone amputations^25^^,^^26^. Due to the lack of previous research, the results related to re-experimentation as the most relevant symptom of PTSD, as well as the gender differences found in this symptom (more prevalent in women than men), cannot be compared. Anyway, the higher rate of re-experimentation found in women is in line with other studies on post-traumatic stress, more prevalent in women than in men^43^.

A relevant result of this study is the impact of an amputation on adjustment to daily life, specifically in terms of labour and leisure. The effect of amputations on quality of life have been previously examined^30^ but the specific domains affected have not been analysed. Furthermore, relevant gender differences have been found in leisure activity, with higher maladjustment in men than women, as it was hypothesized. This result is similar to that found in the study of Ali and Haider^36^, who authors have related the higher rate of maladjustment found in men with their more active previous lifestyles compared to women.

In this study, two out of three workers with amputations experienced phantom limb pain. This relevant rate is in line with the results of the study of Colquhoun et al.^14^, carried out with amputations, regardless of their cause. According to our results, the phantom limb syndrome is also highly prevalent in workers with amputations, without gender differences. Therefore, this phenomenon should be considered when studying work amputations.

Regarding suicidal ideation, our results are much lower than those found in previous studies^27^^,^^28^. Moreover, no suicide attempts were found, in contrast to other studies^27^^,^^29^. This difference is probably due to the use of the Columbia Scale Screening for Suicidal Ideation, which only explores the presence of suicidal behaviours during the last month. Consequently, more research exploring suicidal behaviours since the accident happened is needed.

This study has a number of limitations. First, due to its exploratory and descriptive nature, the specific causal role of amputation in the development of symptoms cannot be established. Future research should explore this phenomenon by including comparison groups and taking a longitudinal perspective. Second, this study only included workers in Navarra, Spain, who agreed to be interviewed. This bias prevents generalizing the results obtained to all workers who have suffered an amputation. Future studies should include samples in other settings and countries. Third, the results of this study must be considered cautiously due to the limited sample size. Although they are promising, this is a preliminary study that sets the guidelines for further investigation. Fourth, a larger sample of women would be necessary to compare the results according to gender. The small sample size, mainly in the case of women, may have limited the statistical power to find significant differences. Finally, this study did not consider other personal and work-related circumstances surrounding the accident that led to the amputation, beyond those already included in the self-reports and the interview. A complementary qualitative approach may improve future studies. These limitations might influence the results, and future research should consider them.

In conclusion, this study determined the socio- demographic, occupational and psychosocial characteristics and type of amputation of people affected by occupational amputations. The results highlight the presence of specific psychosocial consequences in these types of workers. In addition, differences between men and women were explored, with more cognitive re-experimentation in women, and more maladjustment in men. In summary, the study shows the relevance of this topic, which has been scarcely studied. This is a promising field of study for future research on this topic.

## Data Availability

Data cannot be shared for legal and ethical reasons, due to the nature of data gathered. In some cases, access will be provided under restrictions to protect confidential information.
